# Functional Characterization of a Newly Identified Group B Streptococcus Pullulanase Eliciting Antibodies Able to Prevent Alpha-Glucans Degradation

**DOI:** 10.1371/journal.pone.0003787

**Published:** 2008-11-21

**Authors:** Isabella Santi, Alfredo Pezzicoli, Mattia Bosello, Francesco Berti, Massimo Mariani, John L. Telford, Guido Grandi, Marco Soriani

**Affiliations:** Novartis Vaccines and Diagnostics Srl, Siena, Italy; University of British Columbia, Canada

## Abstract

Streptococcal pullulanases have been recently proposed as key components of the metabolic machinery involved in bacterial adaptation to host niches. By sequence analysis of the Group B *Streptococcus* (GBS) genome we found a novel putative surface exposed protein with pullulanase activity. We named such a protein SAP. The *sap* gene is highly conserved among GBS strains and homologous genes, such as PulA and SpuA, have been described in other pathogenic streptococci. The SAP protein contains two N-terminal carbohydrate-binding motifs, followed by a catalytic domain and a C-terminal LPXTG cell wall-anchoring domain. *In vitro* analysis revealed that the recombinant form of SAP is able to degrade α-glucan polysaccharides, such as pullulan, glycogen and starch. Moreover, NMR analysis showed that SAP acts as a type I pullulanase. Studies performed on whole bacteria indicated that the presence of α-glucan polysaccharides in culture medium up-regulated the expression of SAP on bacterial surface as confirmed by FACS analysis and confocal imaging. Deletion of the *sap* gene resulted in a reduced capacity of bacteria to grow in medium containing pullulan or glycogen, but not glucose or maltose, confirming the pivotal role of SAP in GBS metabolism of α-glucans. As reported for other streptococcal pullulanases, we found specific anti-SAP antibodies in human sera from healthy volunteers. Investigation of the functional role of anti-SAP antibodies revealed that incubation of GBS in the presence of sera from animals immunized with SAP reduced the capacity of the bacterium to degrade pullulan. Of interest, anti-SAP sera, although to a lower extent, also inhibited Group A Streptococcus pullulanase activity. These data open new perspectives on the possibility to use SAP as a potential vaccine component inducing functional cross-reacting antibodies interfering with streptococcal infections.

## Introduction

The use of carbon sources is essential to the ability of bacteria to colonize the host and potentially cause disease in humans. In particular, highly polymerized α-glucan polysaccharides, such as starch and glycogen, are most likely to be found in environmental niches. Indeed, it is known that dietary-derived starches are very abundant in the human colon [1], [2], [3], while glycogen is deposited in large amount in the vaginal ephitelium during times of high estrogen availability [4], [5]. Recent reports using *in vivo* models of colonization showed a correlation between the expression of proteins involved in sugars metabolism and virulence. For example, the malto-oligosaccharide/maltodextrin–binding component of the Group A *streptococcus* malto-oligosaccharide ABC transporter has been shown to be directly involved in virulence in a mouse model of oropharynx infection [6]. More recently, Shelburne *et al.* demonstrated that in human saliva the transcript levels of several GAS carbohydrate utilization proteins other than glucose are highly expressed [7]. In addition, a signature-tagged mutagenesis study on *S. pneumoniae* (SPN) highlighted that a number of α-glucan–active enzymes seems to be virulence factors in a mouse model of lung infection [8].

Because of the complex structures of highly polymerized α-glucans, bacteria require an appropriate combination of enzymes for de-polymerization to oligo- and monosaccharides. Among these enzymes are ascribed pullulanases. Pullulanases have a glycosidic hydrolase activity towards α-glucan polysaccharides and are considered key extracellular components in bacterial metabolism. GAS and *Streptococcus pneumoniae* (SPN) pullulanases, named PulA and SpuA respectively, have been recently described [9], [10]. They are anchored to the cell wall at their C termini by an LPXTG motif and possess a modular structure harboring a carbohydrate binding motif belonging to family 41 (CBM41) well distinct from the catalytic domain (CD) [11]. CBMs are currently classified into 47 families on the basis of amino acid sequence [12]. In particular, family 41 in the CBM classification was identified for the first time in a pullulanase enzyme of the marine bacterium *Thermotoga maritime* and it shares a high specificity for α-glucans. Of interest, PulA has been described to have multifunctional activities as the capability to hydrolyze pullulan, a linear polysaccharide of maltotriosyl repeating units linked by α-(1,6) glycosidic linkage [9], [13] and to act as a strepadhesin able to bind to thyroglobulin, submaxillar mucin, fetuin, and asialofetuin [9]. PulA expression is up-regulated by Mga and down-regulated by Rgg, both of which are central transcriptional regulators of *S. pyogenes* gene expression [13]. In addition, it has been recently reported that the recombinant forms of PulA and SpuA CBMs showed high affinity for glycogen-rich alveolar type II cells [10].

Group B *streptococcus* (GBS) is an extracellular mucosal pathogen causing neonatal meningitis and invasive diseases in non-pregnant adults. GBS colonizes the lower gastrointestinal and genital tracts of healthy adults, as approximately 20–30% of healthy women are colonized rectovaginally with GBS [14]. To date, the mechanisms underlying the capacity of GBS to use carbon sources available at site of colonization are largely undefined. By sequence analysis of the GBS genomes, we discovered a novel surface exposed α-glucan degrading-enzyme belonging to the streptococcal family of pullulanase (SAP). Functional characterization of SAP revealed that the protein is immunogenic in humans and that sera from SAP immunized animals are able to reduce the capacity of SAP to degrade α-glucans. Of particular interest, anti-SAP sera were also impairing GAS pullulanase activity. These evidences may draw up the basis for new strategies for preventing the use of environmentally available complex carbohydrates by streptococci.

## Results

### Identification and genomic analysis of SAP

**Figure 1:**
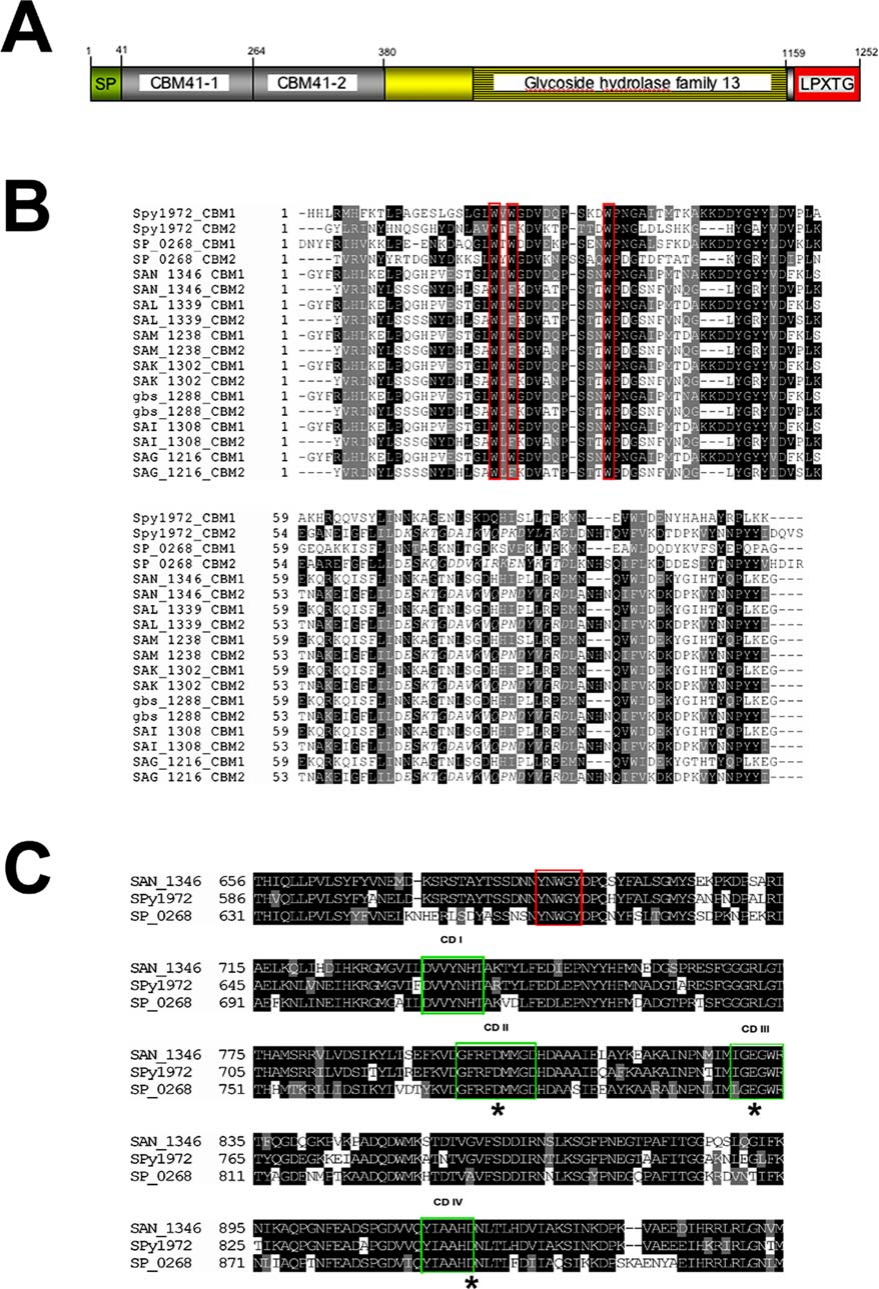
Modular organization of the SAP protein from the COH1 strain. (A) In green the signal peptide sequence. In gray the two tandems CBM41, in yellow the catalytic domain (glycoside hydrolase family 13) and in red the C-terminal LPKTG cell-wall anchoring motif. (B) Sequence alignments of individual CBM41s from GBS COH1 (SAN_1346), 515 (SAL_1339), CJB111 (SAM_1238), A909 (SAK_1302), NEM (gbs_1288), H36B (SAI_1308), 2603 V/R (SAG_1216) strains, GAS SF370 strain (Spy_1972) and SPN TIGR4 strain (SP_0268). The conserved residues present in the α-glucan binding site are boxed in red. In italics is indicated the fibronectin type III repeat. (C) Sequence alignment of the putative pullulanase catalytic domains between GBS COH1 strain (SAN_1346), GAS SF370 strain (Spy_1972) and SPN TIGR4 strain (SP_0268). The four conserved regions designated CDI, CDII, CDIII and CDIV form the catalytic domain and are boxed in green. Indicated with an asterisk are the amino acids forming the putative catalytic triad Asp802-Glu831-Asp919. The YNWGY sequence motif is marked with a red box.

The increasing interest on streptococcal metabolism of complex host-derived carbohydrates supported by recent studies on the involvement of metabolic genes in Group A *streptococcus* (GAS) infections [15], led us to investigate the presence in GBS of surface-associated genes with putative α-glucan hydrolase activity. *In silico* analysis of recently sequenced GBS genomes [16], [17], [18] revealed the presence of a gene (3759 bp) encoding a protein of 1252 amino acid sharing a multimodular architecture comprising a leader peptide (residues 1–41) containing a YSIRK-G/S-like motif [19]. A tandem of putative carbohydrate binding motifs (CBMs) and a glycoside hydrolase catalytic module were also identified (Fig. 1A). On this basis, we named this new protein *Streptococcus agalactiae* pullulanase (SAP). The *sap* gene is present in all sequenced GBS strains and the protein is highly conserved, with an amino acid identity ranges between 98 and 100%. SAP repeated CBMs bore ∼20% amino acid sequence identity to family 41 CBM found in a secreted *Termotoga maritime* pullulanase. CBM41 binds tightly to α-glucan polysaccharide comprising primarily α-(1,4) glycosidic linkages [10]. Of interest, we found that CBMs also shared ∼66% and ∼54% identity to CBMs described in GAS (Spy1972, PulA) and *Streptococcus pneumoniae* (SPN) (SP0268, SpuA) pullulanases respectively (Fig. 1B). Of interest, the second CBM also contains a putative fibronectin-like domain (Fig. 1B, aminoacids in italics). The anchoring domain of SAP to the peptidoglycan is formed by the consensus LPKTG sequence, followed by a hydrophobic transmembrane segment and a charged C-terminal tail. Analysis of the SAP amino acid sequence revealed the presence of an YNWGY motif, common to pullulanases (Fig. 1 C, red box). As shown in Fig. 1C, the catalytic domain is composed by four regions (aa 717 to 723, aa 781 to 789, aa 812 to 817, and aa 897 to 903) conserved among streptococcal pullulanase enzymes (Fig. 1C, green boxes). The putative catalytic triad Asp_802_-Glu_831_-Asp_919_ was identified inside each of the catalytic domains (Fig. 1C). Moreover, we identified a tryptophan residue and a tyrosine residue, in regions III and IV respectively, as found in other pullulanases [20].

### The recombinant form of SAP shows a specific pullulanase enzymatic activity

**Figure 2:**
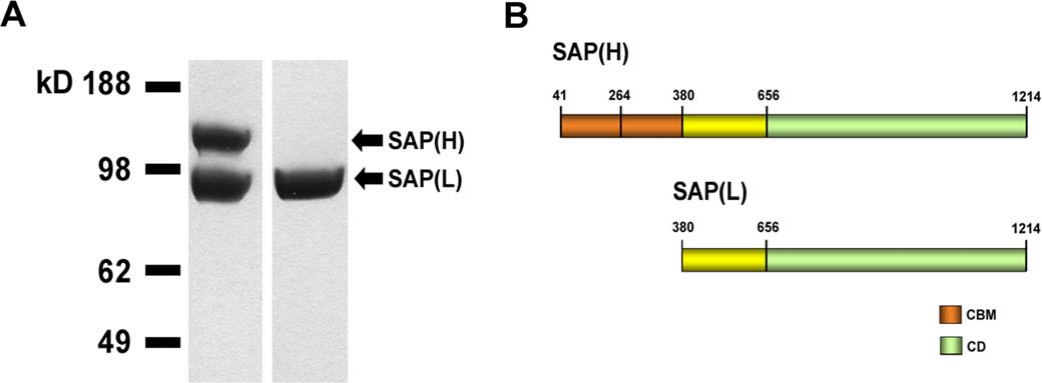
Expression of SAP recombinant protein. (A) SDS-PAGE of the mixture of the high and low molecular weight forms of SAP (left lane) as obtained after affinity chromatography and SAP(L) as obtained after anionic exchange chromatography (right lane). (B) Schematic representation of the recombinant form of SAP. Due to the presence of an alternative translation site the protein is expressed in two forms: SAP(H), the full-length form of the enzyme; SAP(L), the truncated form without the CBMs.

The *sap* gene from the COH1 strain, without the signal sequence and the cell-wall anchoring region, was cloned into pET21b(+) expression vector. As shown in Fig. 2A, two main bands of 130 and 98 kDa were observed on SDS-PAGE gel, suggesting that two forms of the protein were being produced in *E. coli*. This is in agreement with previous data reported in the literature [21], [22] and our data (Bombaci *et al.,* unpublished observations) indicating the same protein pattern for recombinant PulA. On the basis of N-terminal sequencing analysis of the low MW form of SAP, which revealed the MKVQPNDYVF motif, we predicted a second putative GTG translational start codon within the COH1 *sap* ORF at position +1036 and a possible Shine-Dalgarno region (5′-AGGAGA-3′) 4 bp upstream of this point. The resulting translation product obtained from this start site yield a smaller protein lacking both CBMs. A mixture of the high and low molecular weight forms of SAP (H+L) was purified by affinity chromatography. Moreover, by anionic exchange chromatography, we were also able to separate SAP (L) from SAP (H+L). Both SAP recombinant preparations were used to demonstrate that a pullulanase enzymatic activity was associated to the protein.

**Figure 3:**
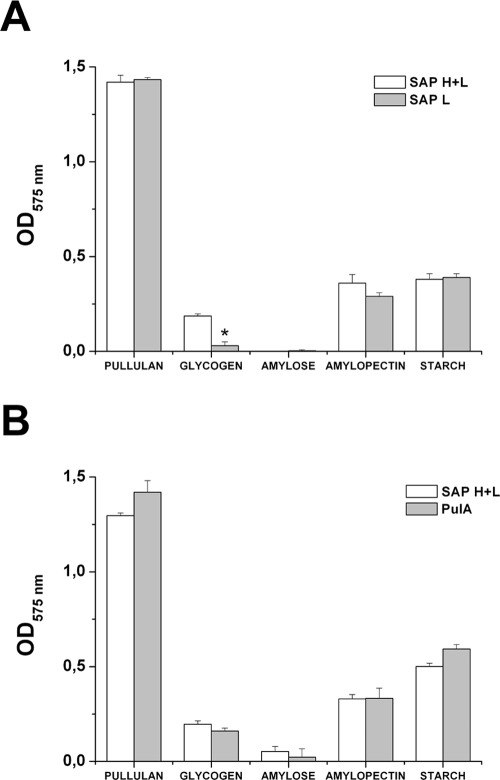
Recombinant SAP enzymatic activity determined by DNS acid assay (A) Determination of the activity of SAP recombinant forms on different a-glucan substrates. The mixtures contained 1% (w/v) of each substrate dissolved in PBS. After incubation of recombinant SAP and a-glucans at 37°C for 1 h, DNS buffer was added and the release of reducing groups was determined by reading the absorption at 575 nm. The same sample without the enzyme was used to correct for non-enzymatic release of reducing sugars. (B) Comparison between recombinant SAP(H+L) and recombinant PulA for the capacity to degrade a-glucans. Experimental protocol as in (A). The data are the mean of 3 independent experiments ± SD. The asterisk indicates a significant difference between values (p<0,01).

The capacity of recombinant SAP to catalyze the degradation of α-glucan polysaccharides was tested by 3,5-dinitrosalicylic (DNS) acid assay (see Experimental procedures). As shown in Fig. 3A, recombinant SAP was active on pullulan, glycogen type IX from bovine liver, amylopectin and starch, in which glucose residues are linked by both α-1-4 and α-1-6 glycosidic linkages. On the contrary, SAP was unable to catabolize amylose, which is a linear glucose polymer carrying exclusively α-1-4 glycosidic linkages. For the specific cleavage of α-1-6 glycosidic linkages, we hypothesize that SAP is likely to be a Type I pullulanase [23].

Of interest, comparison of SAP(H+L) versus SAP(L) preparations, showed that they were both active on pullulan, starch and amylopectin, while only SAP(H+L) was able to degrade glycogen. This finding suggests that the CBM contributes to the specific interaction with glycogen, in agreement with previous reports [8], [10].

In addition, we compared the enzymatic activity of SAP versus GAS pullulanase (PulA) using different carbohydrates as substrates. As shown in Fig. 3B, no statistically differences were observed among SAP(H+L) and PulA for the capacity to degrade pullulan, glycogen, amylopectin and starch. These data postulate that, although the overall sequence conservation is around 60% of identity, pullulanase enzymatic activity well correlates between SAP and PulA.

### SAP is a Type I pullulanase that generates maltotriose residues

**Figure 4:**
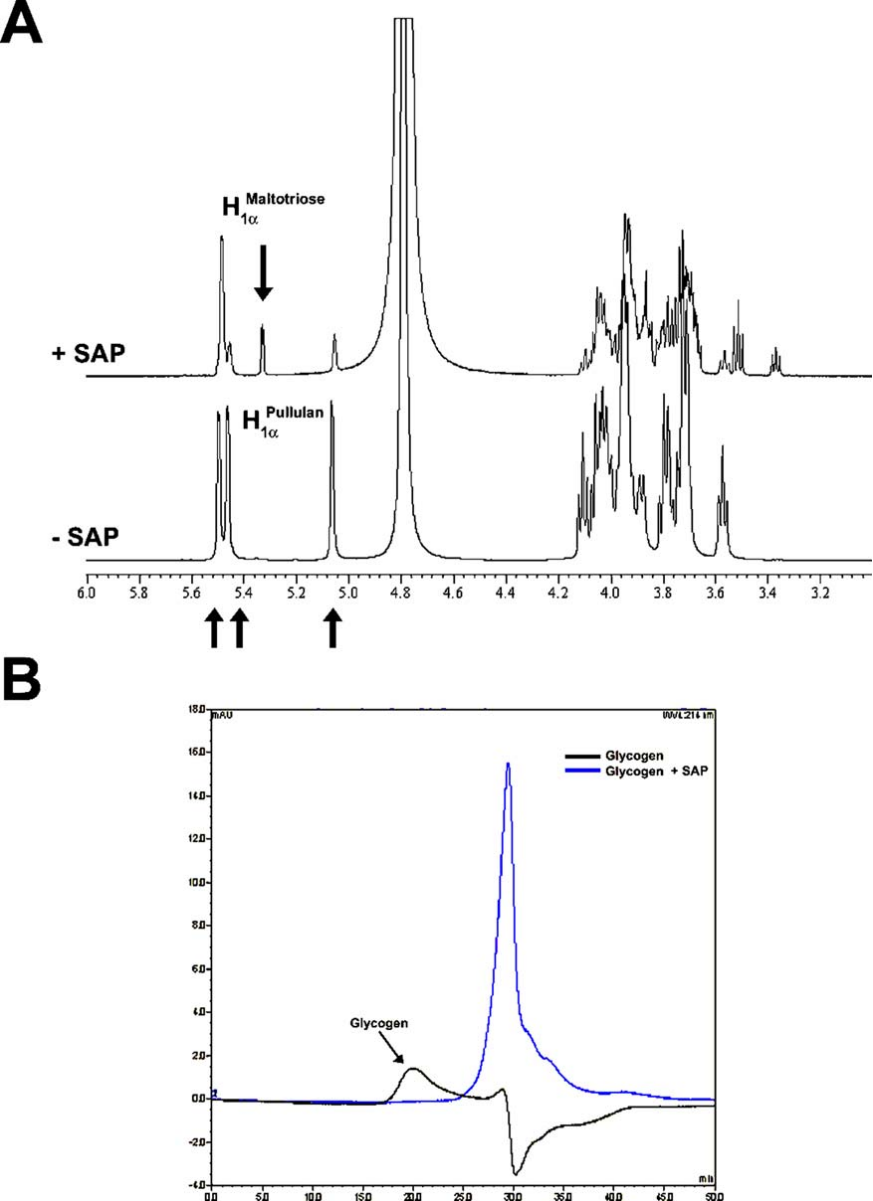
Analysis of SAP(H+L) enzymatic activity on pullulan and glycogen. (A) NMR spectra indicate the generation of maltotriose units after the addition of SAP(H+L) to the reaction mixture containing pullulan. Pullulan NMR spectra were recorded on the native polysaccharide (−SAP) and after the addition of the recombinant enzyme (+SAP). NMR experiments were recorded at 25°C on Bruker Avance 600 MHz spectrometer and using 5-mm probe (Bruker). For details see the Experimental Procedures section. (B) SEC-HPLC analysis indicates that SAP(H+L) is active on glycogen. Two chromatograms were recorded at 214nm, one on the native glycogen polysaccharide (black line) and the other 1 h later the addition of SAP(H+L) (blue line). A gel filtration analytical column with a fractionation range of Mw PEG/PEO 2×10^3^–3×10^5^ Da was used. For details see the Experimental Procedures section.

In order to confirm the classification of SAP as a type I pullulanase we evaluated the modifications of the structure of pullulan after incubation with SAP by NMR spectroscopy. Fig. 4A shows the proton NMR spectra of pullulan incubated in the presence or absence of SAP(H+L). All the signals have been assigned by using ^1^H-^1^H 2D NMR scalar chemical shift correlation spectroscopy, which gave results in agreement with the assignments reported in the literature [24]. The NMR chemical shift of selected signals, particularly looking at the anomeric region (about from 5.8 to 4.5 ppm), has been used to monitor the structural degradation of the polysaccharides. The ^1^H NMR spectrum of pullulan after the addition of SAP(H+L) (Fig. 4A) contains the C1 protons present in the starting material and a new anomeric α-linked signal [H_1α_^(C Maltotriose^] at 5.33 ppm, generated by enzymatic cleavage of the glycosidic bonds. The reducing end β-linked signal [H_1β_^(C Maltotriose^] is not detectable due to the overlapping with the major HDO peak. Since the peak integral ratio between (H_1α_^(C) Maltotriose^+H_1β_^(C) Maltotriose^) and H_1α_^1-4 (A+B) Maltotriose^ is 1∶2, we can conclude that the SAP cleaves α-(1,6) glycosidic linkages between the units A and C generating maltotriose units [25], [26].

Glycogen molecular size distribution before and after the addition of SAP(H+L) was instead determined by size exclusion chromatography. As reported in Fig. 4B, the intensity of glycogen RI signal decreased after 20 min from the addition of SAP(H+L). From these data we can conclude that SAP is also active on glycogen as confirmed by DNS acid assay.

### Alpha-glucans modulates SAP expression on bacterial surface

**Figure 5:**
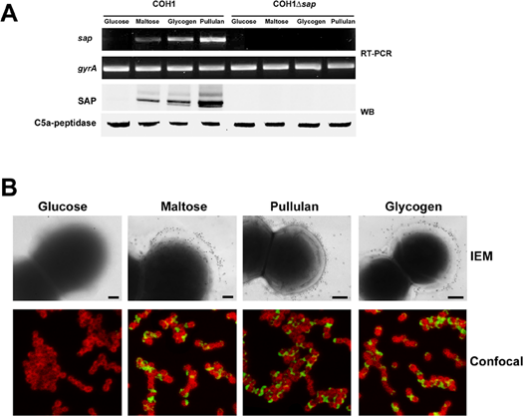
In vivo SAP protein expression is modulated by the presence of α-glucans. (A) RT-PCR and WB analysis of SAP expression in COH1 wt strain and COH1Δsap strain grown in the presence of different sugars. Peptidoglycan-associated protein fraction was separated by 10% (w/v) SDS-PAGE. Blots were overlaid with a mouse anti-SAP polyclonal antibody and stained with HRP-conjugated antibody. (B) Immunogold electron microscopy and confocal imaging of SAP expression in COH1 wild type strain and COH1Δsap strain grown as in (A). For IEM, fixed bacteria were incubated with an anti-SAP serum and then labeled with secondary antibody conjugated to 10-nm gold particles. Scale bars 200 nm. In confocal imaging experiments, bacteria were stained with mouse polyclonal anti-capsular type III antibodies (red) and the SAP protein with rabbit polyclonal anti-SAP antibodies (green). Magnification, ×100.

We observed that when GBS was grown in THB medium, a rich medium normally used to culture GBS in laboratory, SAP was not expressed on bacterial surface (data not shown). Since bacterial pullulanases are known to be regulated by specific carbon sources [27], we hypothesize that the amount of glucose in THB medium (2 g/L) may down-regulate SAP expression. Therefore, expression analysis was performed by using a Complex Medium (CM) to which different α-glucans were added. We investigated the mechanisms of regulation of SAP expression by RT-PCR, Immuno-Electron Microscopy (IEM), confocal microscopy, FACS and Western blotting (WB). As expected, SAP messenger RNA transcript was undetectable when GBS was grown in CM supplemented with glucose (Fig. 5A). On the other hand, a band corresponding to SAP appeared in RNA extracts from bacteria grown in CM plus maltose, pullulan or glycogen. As shown in Fig, 5A, a single band recognized by anti-SAP antibodies was revealed by WB analysis in the mutanolysin-sensitive peptidoglycan protein fraction of bacteria grown in the presence of α-glucans. Control experiments indicated that a cytosolic protein (SAG0274), predicted to be an alpha-glycerophosphate oxidase, was present in bacterial extracts but not in culture supernatants, excluding the presence of contaminants from bacteria debris (data not shown). In addition, FACS analysis (Table 1), IEM and confocal imaging (Fig. 5B) also confirmed that SAP expression on GBS surface is α-glucans dependent. To confirm the specificity of the assays used, we constructed in COH1 strain a *sap* deletion mutant. As expected, the mutant strain was negative for SAP expression as confirmed by RT-PCR (Fig. 5A), WB analysis (Fig. 5A) and FACS analysis (Table 1).

**Table 1:** Exposure of SAP on bacterial surface in the presence of different carbohydrates.

	Glucose	Maltose	Pullulan	Glycogen
COH1	7*	56	232	255
COH1Δ*sap*	0	0	0	0
COH1-13	62	308	453	452

* Numbers indicate the delta mean of fluorescence relative to bacteria incubated with a SAP immune serum versus bacteria incubated with a pre-immune serum.

### A SAP deficient mutant strain shows an impaired capacity to grow in pullulan and glycogen containing complex medium

**Figure 6:**
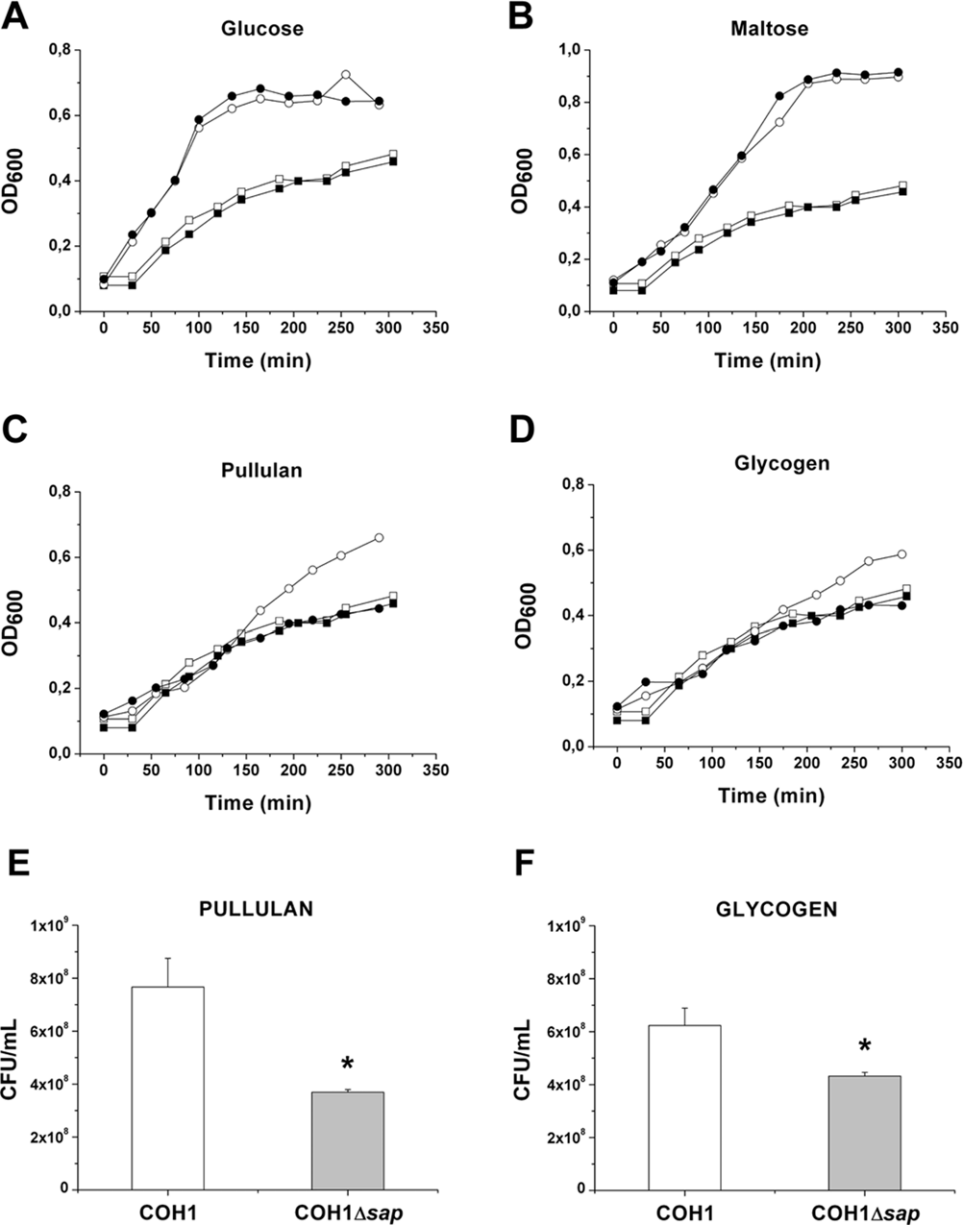
The capacity of GBS to grown in pullulan and glycogen depends on SAP expression. The graphs represent the growth curves relative to GBS COH1 wild type strain and COH1Δsap mutant strain grown in complex medium alone or with the addition of glucose (A), maltose (B), pullulan (C) and glycogen (D). White circles indicate the COH1 wild type strain incubated in the presence of sugars, while white squares the same strain incubated in complex medium alone. Black circles represent the COH1Δsap strain grown in complex medium supplemented with sugars, while black squares are relative to the same strain grown in complex medium alone. A typical experiment, out of 4 performed giving identical results, is shown. (E–F) Comparison of CFU/ml recovered after growing GBS COH1 wild type and COH1Δsap for 3 h in the presence of pullulan (E) or glycogen (F). The data are the mean of 3 independent experiments ± SD. The asterisk indicates a significant difference between values (p<0,01).

To investigate whether SAP enzymatic activity is essential for bacterial replication in the presence of α-glucans, we compared COH1 wild type strain versus COH1Δ*sap* strain for the ability to grow in CM supplemented with different carbohydrates. As expected no growth differences were observed among these strains when glucose or maltose, that are not pullulanase substrates, were added to the CM (Fig. 6A and B). On the other hand, the presence of pullulan or glycogen in CM while did not affect the capacity of the *sap* mutant strain to replicate, increased the growth rate of the wild type strain (Fig. 6C and D).

**Figure 7:**
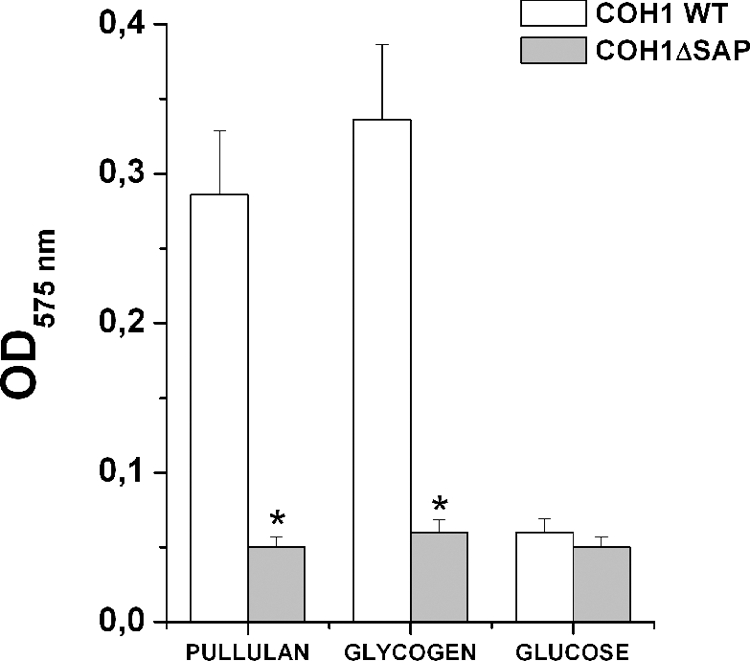
Detection of pullulanase activity in GBS total extracts. Bacteria grown in a complex medium supplemented with the indicated sugars were used to prepare total extracts. Bacterial extracts were then incubated with pullulan and pullulanase enzymatic activity measured by DNS acid assay. The asterisks indicate a significant difference between the activity of the wild type strain versus the mutant strain derived extracts (p<0,01). The data are the mean of 3 independent experiments ± SD.

In order to confirm that the capacity of GBS to hydrolyze α-glucans is associated to an a increased expression of SAP, we compared a total protein extract derived from COH1 *wild type* and COH1Δ*sap* strains grown in the presence of different carbohydrates, for the ability to degrade pullulan. As shown in the Fig. 7, pullulanase activity was only observed in the protein extracts of COH1 *wild type* grown in the presence of sugars inducing SAP expression, such as pullulan and glycogen. These findings suggest that GBS utilize α-glucans as a carbon energy source and that pullulanase is indispensable to this activity.

### SAP is recognized by human sera

**Figure 8:**
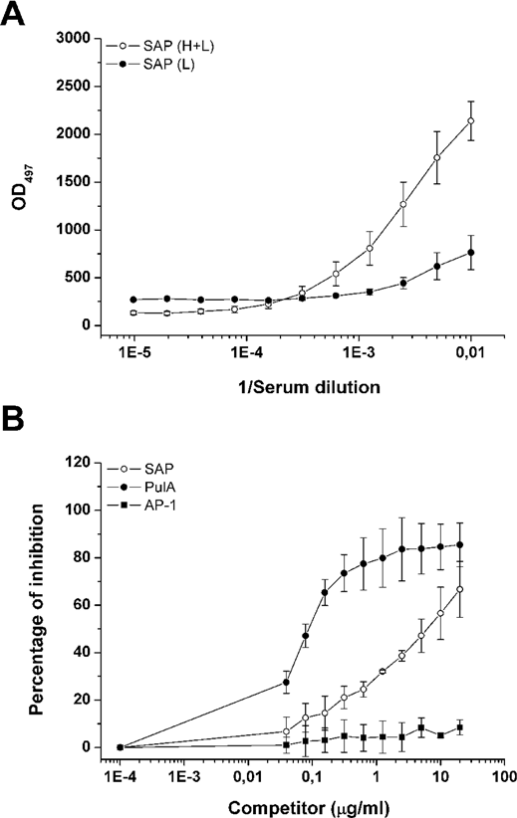
Human sera specifically recognize SAP. (A) Immuno-reactivity of human sera towards recombinant SAP(H+L) and SAP(L). The data were obtained by ELISA and represent the mean±SD of 4 human sera. (B) Results of competitive-inhibition ELISA demonstrating antigenic specificity of human antibodies reacting with plates coated with recombinant SAP. Percent inhibition of binding of human serum by each inhibiting antigen was determined by comparison of absorbance at 492 nm in the presence and absence of inhibitor. White circle labels indicate the mean±SD of the % inhibition by SAP of 4 human sera. Black square labels indicate % inhibition by an unrelated GBS protein (AP-1).

Recent reports revealed that both sera from patients with GAS and SPN infections contained antibodies reactive with pullulanases [28], [29]. In order to assess whether human sera recognized recombinant SAP, we tested 4 sera from normal healthy volunteers. By quantitative ELISA we found that all sera tested showed antibody titers against SAP(H+L) and that IgG concentrations were in a range of 54.8–116.7 µg/ml, with a geometric mean concentration of 76.7±31.9 µg/ml. Of interest, antibody titers against SAP(L) were lower compared to SAP(H+L) (Fig. 8A). The specificity of the assay was confirmed by competitive ELISA using the purified recombinant SAP protein as an inhibiting antigen (Fig. 8B). The addition of an unrelated recombinant GBS surface protein did not inhibit antibody binding to the SAP protein in this assay (Fig. 8B). These findings other than indicating the specificity of the antibody response towards SAP, suggest that the CBMs might be important for the immunogenicity of the protein.

### Anti-SAP antibodies block SAP and PulA enzymatic activity

**Figure 9:**
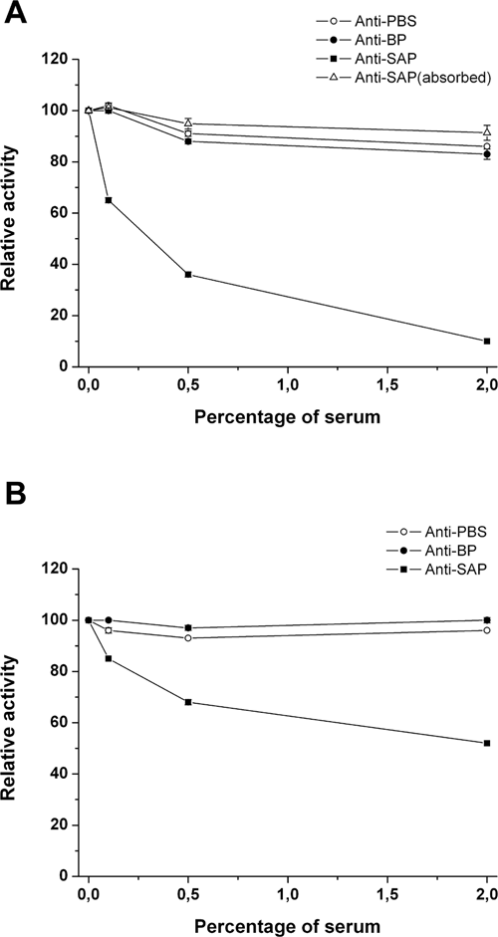
Anti-SAP antibodies block SAP and PulA enzymatic activity. (A) COH1 strain was grown in pullulan and assayed for the capacity to degrade pullulan in the presence of different anti-sera. The effect of specific anti-sera was tested in a dose range between 0.5 and 2%. White circles indicate the effect of a mouse anti-PBS serum; black circles indicate the effect of an antiserum from an unrelated surface-associated protein; black squares indicate the effect of a mouse anti-SAP serum; white triangles indicate the effect of a mouse anti-SAP serum absorbed to a CNBR resin coated with SAP. (B) as in (A) except for testing the inhibitory activity of an anti-SAP serum on GAS SF370 strain.

In order to test whether the immunoglobulin-mediated response towards SAP impaired bacterial metabolic activity versus α-glucans, we tested by DNS acid assay the capacity of mouse and rabbit anti-SAP sera to prevent GBS pullulanase activity. We performed dose-dependent experiments incubating SAP-expressing bacteria with different sera dilutions in a range between 0.1–2%. As shown in Fig. 9, we observed that a SAP mouse antiserum was able to block in a dose dependent fashion the ability of GBS COH1 strain to degrade pullulan up to 80% of the initial activity (Fig. 9A). As expected, the addition in the assay of two unrelated sera did not inhibit GBS SAP activity (Fig. 9A). Similar results were obtained performing the experiments using glycogen as a substrate (data not shown). Depletion of specific anti-SAP antibodies by absorbing the anti-SAP serum to a CNBR resin coated with recombinant SAP, resulted in no inhibition of GBS capacity to catabolize pullulan (Fig. 9A). As a control, the absorbed anti-SAP serum lost the capacity to recognize the recombinant form of SAP in immunoblotting assay (data not shown). These data clearly postulate that anti-SAP antibodies mediate *in vivo* prevention of GBS pullunase activity. The attempt to reduce SAP enzymatic activity by adding to bacteria human sera containing anti-SAP antibodies (up to a concentration of 10%) or anti-SAP antibodies purified from human sera was unsuccessful (data not shown). We hypothesize that the quantity and quality of anti-SAP antibodies derived from adult healthy volunteers that have been in contact with GBS, is not sufficient for our *in vitro* assay. Unfortunately, no sera from convalescent patients or with GBS invasive disease are at the moment available in our laboratory.

Since SAP CBM appears to be a very immunogenic domain (Fig. 8A) and that it is highly homologous among streptococcal pullulanases, we decided to test whether anti-SAP specific sera were able to reduce the capacity of GAS to catabolize pullulan. For these experiments we used GAS SF370 strain, which in the presence of pullulan expresses PulA, as demonstrated by both Western Blotting and FACS analysis (data not shown). As shown in Fig. 9B, we observed that a SAP antiserum was able to reduce the activity of GAS to catabolize pullulan, up to 50% of the initial activity. These data confirm our hypothesis that the activity of anti-pullulanase antibodies may be cross-species.

## Discussion

The increasing need of new vaccine-based preventive strategies replacing the antibiotic prophylaxis used for eradicating GBS colonization of the genital tract of pregnant women, has recently led to identification of antigens conferring a broad protection in mice [30]. The discovery of novel immunogenic virulence factors has also opened new perspectives to tackle GBS-associated infections [31], [32]. In this context, we considered of importance the understanding of GBS genes involved in adaptive metabolism of the bacterium. Indeed, the mechanisms underlying the capacity of GBS to use complex carbon sources available at site of colonization are largely undefined. Several lines of evidence are now indicating that degradation of complex host-derived carbohydrates is crucial to bacterial virulence. In particular, analysis of the transcriptome of GAS in a mouse soft tissue infection model developed by J. Musser's group [15], identified a series of genes highly expressed during adaptive metabolic responses triggered by nutrient signals and hypoxic/acidic conditions in the host. Of interest, among them were identified genes related to amino acid and maltodextrin utilization such as PulA. Indeed, GAS metabolism of complex host-derived carbohydrates may be particularly important during soft tissue infections because of abundant host glycoproteins and host cell contents released during cell lysis. GAS allows transcription of carbohydrate utilization genes and virulence factors also in other low-glucose environmental conditions such as those found in human oropharynx and saliva [15]. In agreement with our findings, it has been recently demonstrated that GAS pullulanase is up regulated in bacteria grown in human saliva, where glucose levels are low, compared to the growth in nutrient rich medium [7]. On this basis, we hypothesize that similar expression patterns may be induced by GBS during colonization of lower gastro-intestinal and female genital tracts, known to be poor in glucose but rich in α-glucans [2], [4]. Our data indicate that SAP-expressing GBS strains actively degrade glycogen and that the recombinant form of SAP lacking both CBMs loses activity versus this substrate. Based on these evidence, we propose that SAP may have a role *in vivo* during the establishment of vaginal colonization and confirms the specificity of CBMs for glycogen [10]. Moreover, *in silico* analysis of GBS available genomes revealed that SAP is the only surface associated protein containing glycosidic domains (Santi *et al.,* unpublished results) and since SAP appears to be the only enzyme expressed by GBS capable to catabolize α-glucans, we suggest that this enzyme may be vital for GBS permanence in environmental niches poor in glucose. We are currently testing this hypothesis, and preliminary data have shown that deletion of the *sap* gene reduces the capacity of GBS to colonize the vagina in a mouse model of infection (Pezzicoli *et al.*, unpublished results).

The importance of α-glucans metabolizing enzymes in streptococcal adaptation to the host is further highlighted by recent reports revealing they are immunogenic. Indeed, 81% of convalescent-phase sera from patients with invasive GAS infections had antibodies reactive with PulA [28] and sera taken from patient recovering from pneumococcal infections had high titers against SpuA [29]. In agreement to this, we found that also SAP is immunogenic in human sera, supporting the hypothesis that this enzyme is expressed *in vivo* during GBS infections. However, to our knowledge the functionality of streptococcal pullulanase-induced antibody-mediated immune response has not been yet addressed. The fact that incubation of bacteria with an anti-SAP serum reduces GBS pullulanase activity, led us to hypothesize that *in vivo* antibody-mediated immune response towards SAP might affect GBS adaptive metabolism resulting in a decreased capacity to survive in the host.

Emerging theories on human-microbe mutualism suggest that the mechanisms that underlie microbial community structure and host–symbiont relationships should be considered for planning prevention strategies for human health. Indeed, as recently proposed by David A. Relman [33], it should be investigated the role of microbial communities, and not just individual species, as pathogens. In this perspective, reduction of the fitness of pathogens by affecting their metabolic activity towards essential nutrients may be more effective than a general bactericidal activity, as the one offered by an antibiotic treatment. Vaccines able to specifically prevent infection from multiple microorganisms are highly desirable. In this context, our finding that anti-SAP sera other than preventing GBS catabolism of pullulan, significantly reduce pullulanase activity in a GAS strain expressing PulA is of extreme importance. In particular, we hypothesize that the immunization of individuals with SAP will raise antibodies, which by impairing the metabolic activity of pathogenic streptococci might shift the equilibrium that regulates the colonized human niches in favor of the commensal population.

In conclusion, the evidence reported in this paper may draw up the basis for preventing streptococcal infections by using immunogenic metabolic enzymes as target molecules for vaccine development. The fact that, at least for pathogenic streptococci, such enzymes are well conserved opens new perspectives in the development of strategies preventing infections from multiple species.

## Materials and Methods

### Sequence analysis

The alignment of SAP protein encoded by *sap* gene from 2603 V/R (TIGR Accession SAG_1216), 515 Ia (SAL_1339), NEM316 (gbs1288), H36B (SAI_1308), CJB111 (SAM_1238), A909 (SAK_1302) and COH1 (SAN_1346) strain as well as SpuA (SP_0268) and PulA (Spy_1972) was performed using ClustalW [34].

### Bacterial strains and growth conditions

*S*. *agalactiae* strains COH1 serotype III was used in this study. *Escherichia coli* DH5α and DH10BT1 were used for cloning purposes and *E. coli* BL21 (λDE3) for expression of SAP fusion protein. *S*. *agalactiae* was cultivated at 37°C in Todd-Hewitt broth (THB) up to desired OD_600_. *E*. *coli* was grown in Luria-Bertani broth (LB); *E. coli* clones carrying the plasmids pJRS233 or pET21(b)+ and derivates were grown in the presence erythromycin (400 µg/mL) or ampicillin (100 µg/mL), respectively. The complex medium (CM, 10 g/l proteose peptone, 5 g/l trypticase peptone, 5 g/l yeast extract, 2.5 g/l KCl, 1mM Urea, 1mM Arginine, pH 7.0) was used for GBS growth with defined carbon sources. The sugar concentrations were 1% final. To evaluate growth in CM, GBS was initially grown to log phase (OD_600_ 0.3) in THB. The cells were harvested by centrifugation, washed twice in an equivalent volume of phosphate-buffered saline (PBS) and diluted 1 to 50 in CM. Growth was monitored spectrophotometrically at a wavelength of 600 nm.

### SAP recombinant protein expression and purification

In order to express the recombinant form of SAP, the open reading frame of the *sap* gene from *S. agalactiae* COH1 serotype III was used as a template. The construct was amplified by PCR using specific primers GBS5F and GBS5R introducing *Nde*I and *Xho*I restriction enzyme sites (Table 2).

**Table 2:** List of primers used in the study.

Primer	Sequence (5′ to 3′)
**Recombinant protein**	
**GBS5F**	CTAGCTAGCGAAGAAGTAAGTGTTTCTC
**GBS5R**	CCCGCTCGAGATTAGCTTCATTTGTCAGA
**COH1Δ*sap***	
**P1**	CCCGCTCGAGTCATCTACACACGCATTTTTCC
**P2**	TCCAGTTTTTGGCAAGGGAGTATTTTGCAATGTAGATGG
**P3**	TTGCAAAATACTCCCTTGCCAAAAACTGGAGATAAGTCA
**P4**	CCCGCTCGAGTTCCTAATGCTGTCTTCCCAAC

F corresponds to forward primer and R to reverse primer. Restriction sites are underlined.

The PCR products were cloned into the pET21(b)+ vector and the plasmid transformed in *E. coli* BL21 (DE3) cells. BL21 (DE3) cells were grown in LB-Amp (100 µg/ml ampicillin) and induced with IPTG at a final concentration of 1 mM for 3 hours. The resulting biomass was suspended in 0.3 M NaCl, 50 mM Na-PO_4_ buffer, pH 8.0 and cells were lysed by enzymatic digestion. The sample was then loaded onto a His-Trap Ni-Activated Chelating Sepharose FF column (Amersham Biosciences, Milan, Italy) at a flow rate of 5 ml/min. Bound proteins were then eluted from the column by running a gradient from 0 to 50% of 500 mM Imidazole, 0.3 M NaCl, 50 mM Na phosphate buffer, pH 8.0 in 12 CV. The IMAC eluted material was collected in 2.5-ml fractions and those ones containing the SAP-His protein pooled. An anionic exchange chromatography was used to separate the two forms of SAP. The pooled fractions from Ni-IMAC were dialyzed against 30mM TRIS, pH 8.0 and then loaded on to a HiTrap Q HP 5 ml column (GE) to further purify the two forms of recombinant SAP. The purification was achieved by running a gradient from 0 to 50% 1M NaCl in 30mM TRIS, pH 8.0 in 16 CV at 5 ml/min. The collected fractions were analyzed by SDS-PAGE (Criterion™ pre-cast gel, 200V, 55 min) and pooled according to apparent MW. The final preparation of the protein was obtained in PBS, pH 7.4 after dialysis.

### Construction of COH1 sap deletion mutant

The *sap* gene was deleted in *GBS* strain COH1, according to the procedure previously described [35]. The in-frame deletion fragment was obtained by Splicing Overlap Extension (SOE) PCR using the primers P1, P2, P3 and P4 (Table 2). The *Xho*I restriction enzyme cleavage sites were incorporated at the 5′-end of the primer to clone the fragment into the *Xho*I-digested pJRS233 plasmid. After cloning the in frame deletion fragment in pJRS233, the plasmid pJRS233Δ*sap* was obtained.

The plasmid pJRS233Δ*sap* was then transformed into the COH1 strain by electroporation and transformants were selected after growth at 30°C on agar plates containing 1 µg/ml erythromycin. Transformants were then grown at 37°C with erythromycin selection as previously described [36]. Integrant strains were serially passaged for 5 days in liquid medium at 30°C without erythromycin selection to facilitate the excision of plasmid pJRS233Δ*sap*, resulting in the *sap* deletion on the chromosome. Dilutions of the serially passaged cultures were plated onto agar plates, and single colonies were tested for erythromycin sensitivity to confirm the excision of pJRS233Δ*sap.* The resulting strain was named COH1Δ*sap*.

### Bacterial extracts

GBS protein extracts were prepared by growing bacteria up to OD_600_ 0.4 in CM plus sugars, washed in PBS and incubated for 1 h at 37°C in 500 µl of Tris-HCl 50 mM (pH6.8) containing protease inhibitors and 400 U/ml of mutanolysin (SIGMA, MO, USA). The bacterial suspension was then pelleted and the supernatants containing peptidoglycan associated proteins used for western blotting analysis of SAP. In order to prepare GBS extracts relative to the secreted protein fraction, supernatant of bacteria cultures grown to OD_600_ 0.4 were collected. Proteins in 1 ml of supernatant were precipitated with 10% of trichloroaceticacid (TCA) for 1 hr at 4°C. Protein were then pelletted, washed with cold acetone and resuspended in Tris-HCl pH 6,8.

### RT-PCR

COH1 was grown in CM medium plus sugars up to OD_600_ 0.4. Total GBS RNA was isolated using the Rneasy mini kit (Qiagen) according to manufacturer's instructions, except that bacteria were lysed with 100 µl of lysozyme (30 mg/ml) in Tris-EDTA buffer and 2,000 U of mutanolysin, and the mixture was incubated for 15 min at 37°C. Quantification of the transcripts was completed by reverse transcription and semi-quantitative RT-PCR using ImPromII RT (Promega) following manufacturer's instructions. Briefly 2 µg of sample and 0.5 µg of random hexamers were added to a final volume of 5 µl. Samples were incubated in a thermocycler (Biometra) at 70°C for 5 min followed by a quick chill at 4°C. The mixture was used in a 20-µl (total volume) cDNA synthesis reaction mixture comprising 4 µl of Improm-II 5× reaction buffer (Promega), 2.4 µl MgCl_2_ at 25 mM, 2 µl of dNTP mix (each dNTP at 2.5 mM), 0.25 µl of Rnasin RNase inhibitor (Promega) and 1 µl of Improm-II reverse transcriptase. The reaction was performed at 42°C for 60 min. In the negative controls, the reverse transcriptase was substituted with water. 2 µl of cDNA were then added to the PCR reaction consisting of 1× reaction buffer, 200 µM dNTP's, 0.2 µM primer pair, 1 U PlatinumTaq dna polymerase (Invitrogen). GBS Gyrase A (GyrA) was used as an internal housekeeping control. PCR reactions consisted of a 7-min denaturation step 94°C, followed by a variable number of cycles. PCR products were electrophoresed through 2% agarose gels and images were acquired by laser densitometry (Gel-Doc Imaging System).

### NMR analysis

Samples of pullulan (Sigma) and glycogen (Sigma) were prepared by dissolving polysaccaride powder (10 mg) in 0.7 mL of deuterated PBS buffer at pH 7.2 (D_2_O, 99.9% atom D – Aldrich was used) to a uniform concentration. Samples were therefore transferred to 5-mm NMR tubes (Wilmad Glass. Co.). 70 µL of SAP were therefore added to the pullulan and glycogen samples in the NMR tubes. For every sample, two NMR spectra were recorded, the first on the native polysaccharide and the second 1 hour later (incubation at 25°C) after the addition of enzyme. Samples of maltose (Sigma) and maltotriose were also prepared by dissolving 10 mg of powder in 0.7 mL of deuterated PBS buffer at pH 7.2. ^1^H NMR experiments were recorded at 25°C on Bruker Avance 600 MHz spectrometer and using 5-mm probe (Bruker). For data acquisition and processing XWINNMR software package (Bruker) was used. 1-D proton NMR spectra were recorded using a standard one-pulse experiment. 64 scans were collected and averaged, giving a total acquisition time of ca. 10 min. The transmitter was set at the HDO frequency, collecting 32 k data points over a spectral window of 6,000 Hz. ^1^H NMR spectra were obtained in quantitative matter using a total recycle time to ensure a full recovery of each signal (5× Longitudinal Relaxation Time T1). Spectra were Fourier Transformed to 32 k data points after applying a 0.2 Hz line broadening function and referenced relative to the HDO resonance at 4.79 ppm.

### Size Exclusion Chromatography (SEC)-HPLC analysis

Samples of glycogen were prepared by dissolving polysaccaride powder (1 mg) in 0.1 mL of PBS at pH 7.2 to a uniform concentration. Samples were therefore transferred to 1 mL vials (Waters). 10 µL of SAP were therefore added to the glycogen sample. Two chromatograms were recorded, the first on the native polysaccharide and the second 1 hour later (incubation at 25°C) after the addition of enzyme.

A TSK G4000PW (TosoHaas) gel filtration analytical column (7.5 mm×30.0 cm) with a fractionation range of Mw PEG/PEO 2,000–3×10^5^ Da was used. Samples were loaded onto the gel filtration column and eluted isocratically in 100 mM sodium phosphate + 100 mM NaCl buffer pH 7.2 at a flow rate of 0.5 ml min^−1^ for 50 min. The elution was monitored with a Ultimate 3000 Photodiode Array detector (Dionex) coupled with the Ultimate 3000 HPLC system (Dionex). For data acquisition and processing Chromeleon software package (Dionex) was used.

### Fluorescence-activated cell sorter analysis

In order to quantify the exposure of SAP on the bacterial surface, GBS was grown up to OD_600_ 0.4 in CM with 1% sugar, fixed with 1% PFA for 20 min at RT and incubated with mouse anti-SAP serum or mouse anti-PBS serum (negative control) in 0.1% BSA plus 20% of Normal Calf Serum (NCS) for 1 h at 4°C. Bacteria were then washed in PBS containing 0.1% BSA and incubated with the phicoerytrin (PE) conjugated secondary antibodies (Jackson Immuno Research Inc., PA, USA) for 45 min at 4°C. After washing bacteria were resuspended in 200 µl of PBS and analyzed by a FACSscan flow cytometer (Becton Dickinson) by using FlowJo software program.

### Immunogold labeling and electron microscopy

GBS strains COH1 were grown at 37°C up to OD_600_ 0.3 (exponential phase) in CM plus 1% sugars. Bacteria were then centrifuged for 10 min at 3000 rpm (RT), washed and resuspended in 1 ml of PBS. Formvar-carbon-coated nickel grids were floated on drops of GBS suspensions for 5 min. The grids were then fixed in 2% PFA for 5 min, and placed in blocking solution (PBS containing 1% normal rabbit serum and 1% BSA) for 30 min. The grids were then floated on drops of primary antiserum against the SAP protein diluted 1∶20 in blocking solution for 30 min at RT, washed with six drops of blocking solution, and floated on secondary antibody conjugated to 10-nm gold particles diluted 1∶20 in 1% BSA for 30 min. The grids were examined using a TEM GEOL 1200EX II transmission electron microscope.

### Confocal immunofluorescence microscopy

In order to visualize SAP on bacterial surface, COH1 was grown in CM plus sugars up to OD_600_ 0.4 and washed in PBS. Bacterial pellet were fixed in 2% PFA for 20 min at RT and spotted on POLYSINE™ slides (Menzel-Glaser). The slides were then blocked with 3% BSA for 1 h and incubated with a mix of rabbit anti-serotype III capsule and mouse anti-SAP antibodies diluted in 1% BSA for 1 h at RT. Bacteria were then stained with goat anti-mouse and anti-rabbit Alexa Fluor conjugated antibodies (excitation at 488 nm and 568 nm, respectively) (Molecular Probes) for 20 min at RT. Slow Fade reagent kit (Molecular Probes) was then used to mount cover slips. The slides were analysed with a Bio-Rad confocal scanning microscope.

### 3,5-dinitrosalicylic acid (DNS) assay

Pullulanase activity was determined by measuring the enzymatic release of reducing groups from α-glucans by the DNS colorimetric method [37]. The mixtures contained 1% (w/v) pullulan, glycogen type IX, amylose, amylopectin or soluble starch (Sigma) dissolved in PBS (pH 7.0), and appropriately diluted enzyme in a total volume of 500 µL. After incubation at 37°C for 1 h, the reaction was stopped by addition of 1 mL of cold DNS buffer, followed by boiling for 15 min. 330 µL of a 40% potassium sodium tartrate (Rochelle salt) solution was added to each tubes to stabilize the color. The release of reducing groups from α-glucans was determined by reading the absorption at 575 nm of the sample. The same sample without the enzyme was used to correct for non-enzymatic release of reducing sugars.

### Serum-mediated inhibition of GBS and GAS pullulanase activity

GBS and GAS were grown at 37°C up to mid-late exponential phase (OD_600_ 0.6) in THB and THY, respectively. Bacteria were then re-inoculated in CM containing 1% pullulan and grown to log phase (OD_600_ 0.4) to allow the expression of pullulanases on bacterial surface. The cells were then harvested by centrifugation, washed twice with PBS and resuspended in PBS. Bacteria (∼5×10^8^ CFU) were pre-incubated with sera dilutions at 37°C for 15 min, then pullulan was added (1% final) and the incubation prolonged for other 2 hours. Samples were centrifuged and supernatants were used for the determination of reducing sugars by DNS acid assay.
